# DNA Damage Tolerance Pathways in Human Cells: A Potential Therapeutic Target

**DOI:** 10.3389/fonc.2021.822500

**Published:** 2022-02-07

**Authors:** Ashlynn Ai Li Ler, Michael P. Carty

**Affiliations:** ^1^ Biochemistry, School of Biological and Chemical Sciences, The National University of Ireland (NUI) Galway, Galway, Ireland; ^2^ DNA Damage Response Laboratory, Centre for Chromosome Biology, NUI Galway, Galway, Ireland

**Keywords:** DNA damage, DNA damage tolerance pathways, DNA replication, translesion synthesis (TLS), TLS inhibitors, cancer therapeutics

## Abstract

DNA lesions arising from both exogenous and endogenous sources occur frequently in DNA. During DNA replication, the presence of unrepaired DNA damage in the template can arrest replication fork progression, leading to fork collapse, double-strand break formation, and to genome instability. To facilitate completion of replication and prevent the generation of strand breaks, DNA damage tolerance (DDT) pathways play a key role in allowing replication to proceed in the presence of lesions in the template. The two main DDT pathways are translesion synthesis (TLS), which involves the recruitment of specialized TLS polymerases to the site of replication arrest to bypass lesions, and homology-directed damage tolerance, which includes the template switching and fork reversal pathways. With some exceptions, lesion bypass by TLS polymerases is a source of mutagenesis, potentially contributing to the development of cancer. The capacity of TLS polymerases to bypass replication-blocking lesions induced by anti-cancer drugs such as cisplatin can also contribute to tumor chemoresistance. On the other hand, during homology-directed DDT the nascent sister strand is transiently utilised as a template for replication, allowing for error-free lesion bypass. Given the role of DNA damage tolerance pathways in replication, mutagenesis and chemoresistance, a more complete understanding of these pathways can provide avenues for therapeutic exploitation. A number of small molecule inhibitors of TLS polymerase activity have been identified that show synergy with conventional chemotherapeutic agents in killing cancer cells. In this review, we will summarize the major DDT pathways, explore the relationship between damage tolerance and carcinogenesis, and discuss the potential of targeting TLS polymerases as a therapeutic approach.

## Introduction

It is estimated that up to 50,000 DNA lesions can occur per cell in a single day, an average of around 2,000 DNA lesions per cell per hour (1). While the majority of DNA damage is removed by repair pathways, including nucleotide excision repair and base excision repair, prior to cells entering S-phase, lesions can remain in the DNA template during DNA replication. The main DNA polymerases that carry out genomic DNA replication, polymerase δ (Pol δ) on the lagging strand and polymerase ϵ (Pol ϵ) on the leading strand, can both be blocked by DNA damage in the template strand, leading to replication fork stalling, fork collapse, chromosome breakage and genomic instability. To resolve this problem, DNA damage tolerance (DDT) pathways that allow replication of damaged DNA to continue while reducing genomic instability, are present in virtually all organisms (1–4).

## The Main DNA Damage Tolerance Pathways in Eukaryotes

**Figure 1:**
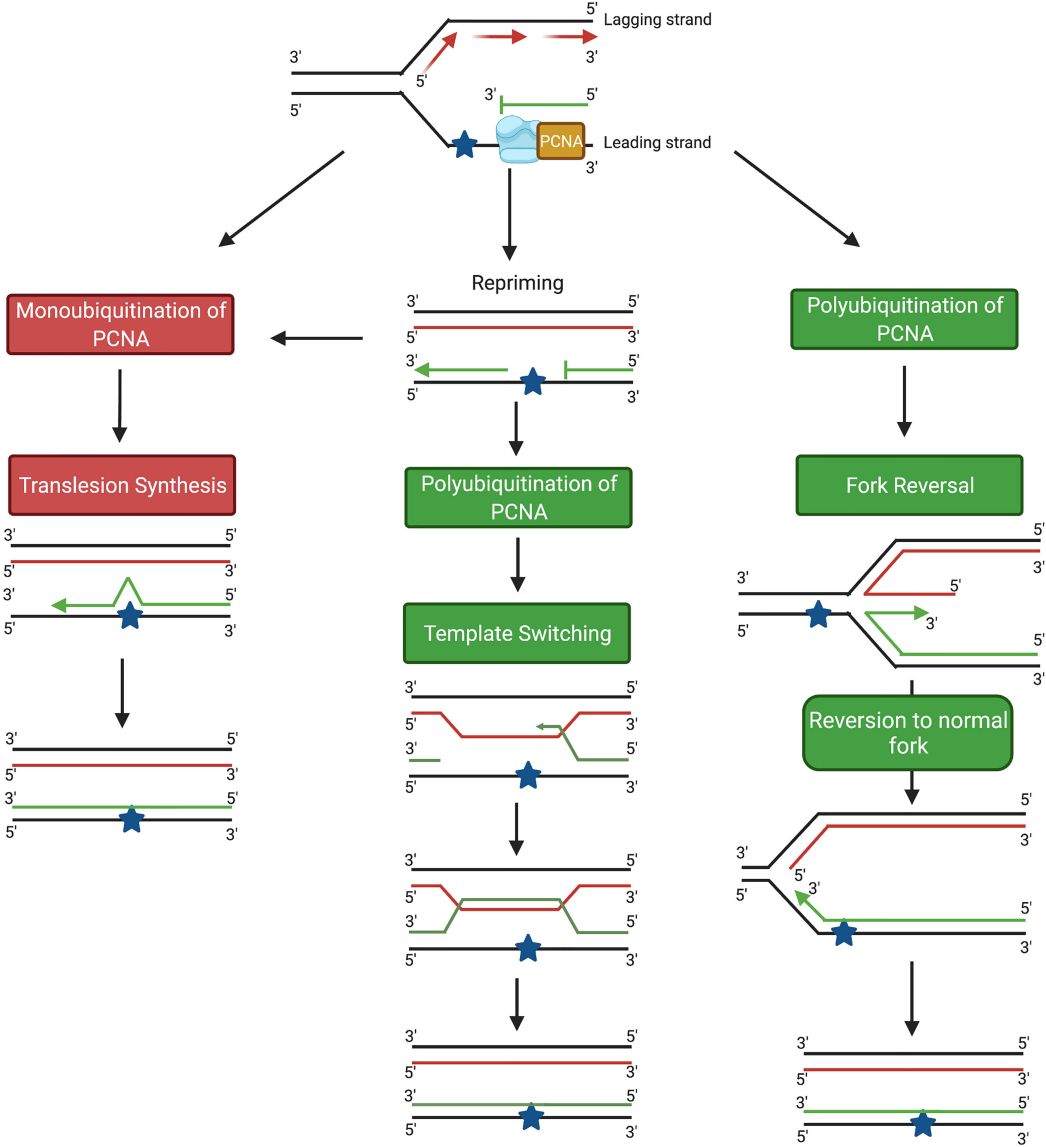
Schematic diagram showing the main DDT pathways in eukaryotic cells. TLS pathways are highlighted in red; homology-based damage tolerance pathways are highlighted in green. Image generated with BioRender.com.

There are two main DDT pathways described in eukaryotic cells, namely (i) translesion synthesis (TLS) and (ii) homology-directed DDT (**Figure 1**). TLS involves the recruitment of specialized TLS DNA polymerases to the arrested replication fork to facilitate lesion bypass, which can take place either directly at the replication fork, or behind the fork by repriming DNA synthesis at daughter strand gaps (DSGs) (**Figure 1**) (5). In response to DNA damage, monoubiquitination of the clamp protein proliferating cell nuclear antigen (PCNA) results in recruitment of the specialized TLS polymerases required to bypass the DNA lesion. Lesion bypass takes place either directly at the site of the arrested fork, or during gap-filling subsequent to replication restart away from the lesion site (6, 7) (**Figure 1**). However, despite some exceptions discussed below, bypass by TLS polymerases contributes to mutagenesis owing to the tendency for base misincorporation opposite lesions (7–9). In fact, the error-prone nature of TLS polymerases has been implicated both in the development of cancer and in promoting chemoresistance in cancer cells (10–12). Hence, TLS is considered an error-prone DDT pathway (13).

In addition to TLS, damage tolerance can also take place through homology-directed DDT, which consists of two pathways, fork reversal (FR) and template switching (TS) (**Figure 1**) (5). Both FR and TS are initiated through the polyubiquitination of PCNA, and involve a temporary switch from the damaged template strand to using the newly-synthesized copy of the complementary strand on a homologous sister chromatid as the template for DNA synthesis. Because an undamaged template is copied, FR and TS are error-free lesion bypass pathways (5). Fork reversal involves formation of a ‘chicken foot’-like DNA structure, allowing the replisome on the arrested nascent strand to gain access to the homologous sister template (14, 15) (**Figure 1**). In contrast, TS occurs following repriming at DSGs generated at lesion sites behind the replication fork (16, 17). TS involves strand invasion, where the newly-synthesized strand from the homologous sister chromatid transiently serves as a template for nascent strand synthesis to allow the replication machinery to bypass the lesion (13, 18) (**Figure 1**).

On a biochemical level, the process of damage tolerance is complex, requiring multiple proteins. While these proteins are potential targets for development of novel cancer therapeutics, a more complete understanding of the molecular genetics, cell biology and biochemistry of damage tolerance is necessary to advance this potential. The present review provides an overview of the main DDT pathways in human cells, and discusses recent advances in targeting these pathways to develop cancer therapeutics.

## Initiation of DNA Damage Tolerance (DDT)

**Figure 2:**
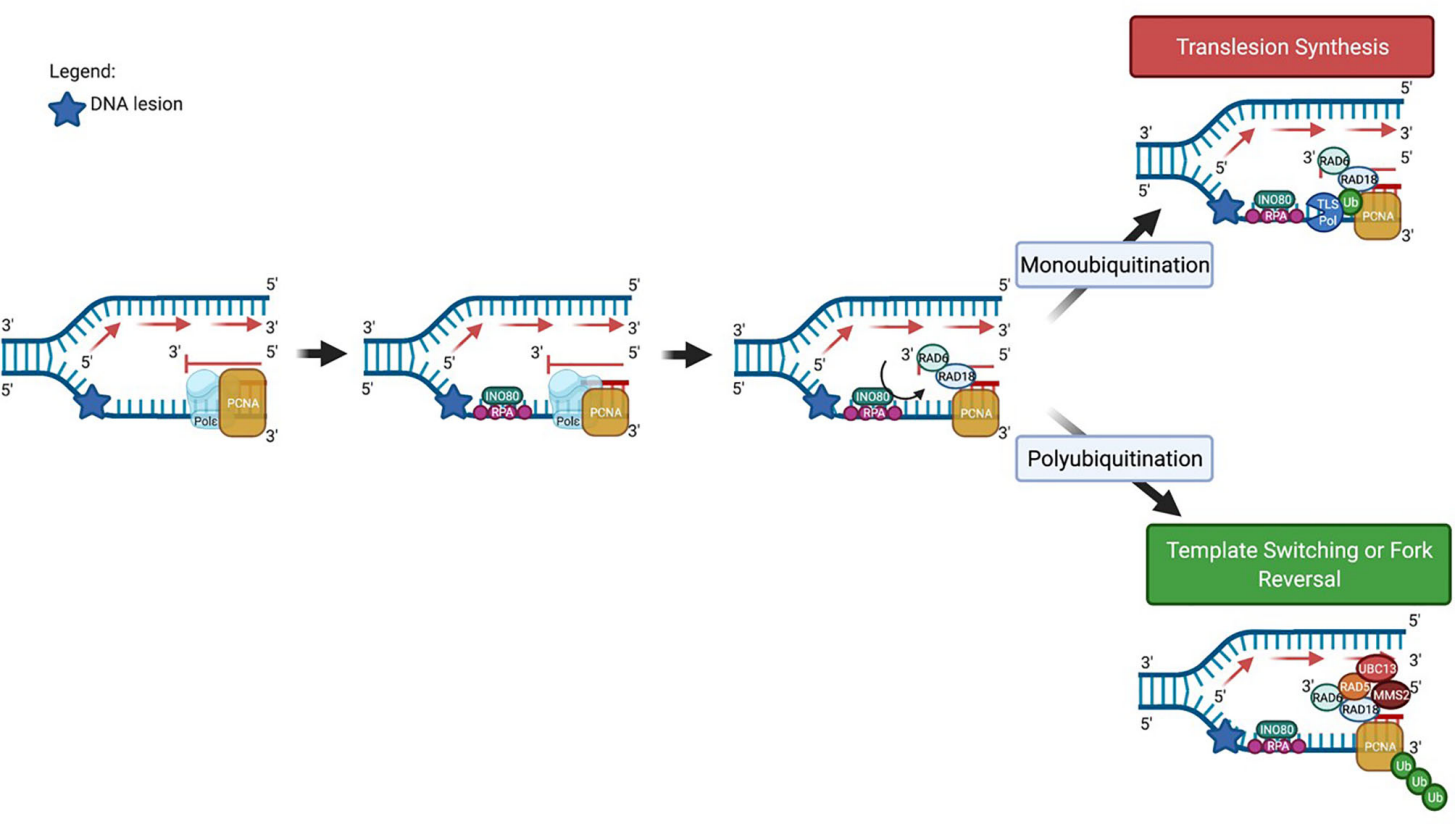
Schematic diagram showing key proteins involved in the initial steps of DNA damage tolerance pathway activation at an arrested replication fork. PCNA, proliferating cell nuclear antigen; RPA, replication protein A; UBC13, Ubiquitin-conjugating enzyme E2 13; MMS2, Ubiquitin-conjugating enzyme variant MMS2. Image generated with BioRender.com.

Both the TLS and TS pathways share common initial steps. Stalling of the replicative DNA polymerase at a DNA lesion site together with ongoing helicase activity at the replication fork generates a region of single-stranded DNA (ssDNA) on the template strand which is bound by replication protein A (RPA). The ssDNA-RPA complex recruits ATRIP, and activates the ataxia telangiectasia and RAD3-related protein (ATR)-dependent replication checkpoint (18–20). At the same time, the chromatin remodelling protein INO80 binds to the stalled replication fork (18, 21–23). This, in conjunction with the RPA-ssDNA complex facilitates the recruitment of the RAD18 E3 ubiquitin ligase to the site of DNA damage (18, 23–26). At the lesion site, RAD18 recruits the E2 ligase RAD6, leading to the formation of the E2-E3 ubiquitinase (18, 25, 27–30) which monoubiquitinates PCNA on K164 (18, 25, 31–33). Monoubiquitination can be facilitated by other E3 ligases such as ring finger protein 8 (RNF8) in conjunction with the E2 ligase, Ubiquitin-conjugating Enzyme H5c (UbcH5c) (34). At this step, the two DDT pathways diverge, with monoubiquitination of PCNA on K164 resulting in the induction of TLS, while polyubiquitination at K164 leads to homology-directed DDT (**Figure 2**).

## Translesion Synthesis

Following monoubiquitination of PCNA, one or more TLS polymerases are recruited to the stalled replication fork. Human TLS polymerases comprise proteins belonging to 4 families: the Y-family (Rev 1, Pol η, Pol ι and Pol κ), the A-family (Pol θ), the B-family (Pol ζ) and the archaeo-eukaryotic primase (AEP) family (PrimPol) (6, 35–38). In Y-family TLS polymerases, the active site that catalyses lesion bypass is located within the conserved N-terminal domain (39, 40), while the variable C-terminal region facilitates recruitment of the protein to stalled forks (39, 40). Y-family TLS polymerases can bind directly to K164-ubiquitinated PCNA through ubiquitin-binding zinc fingers (UBZ) found in Pol η and κ, or to ubiquitin-binding motifs (UBM) present in Pol ι and Rev1 (39). The PCNA-interacting peptide (PIP) boxes on Pol ι, η and κ, and the BRCA1 C-terminus (BRCT) domain in the N-terminal of Rev1, also facilitate the binding of TLS polymerases to PCNA (39, 41, 42).

TLS generally occurs by either a ‘one-polymerase’ mechanism or a ‘multiple-polymerase’ mechanism (35). Upon replication fork stalling in the presence of DNA damage, the replicative polymerase (δ or ϵ) is replaced by a TLS polymerase. Following this step, in the one-polymerase mechanism, a single TLS polymerase inserts nucleotides at the lesion site and continues to extend the replicated DNA strand past the lesion site, and is then replaced again by the replicative polymerase (35). The multiple-polymerase mechanism usually involves two TLS polymerases working in concert, such that one polymerase inserts a nucleotide opposite the lesion site, while the other extends the primer beyond the lesion site (35, 40, 43). Rev1 incorporates a single dCTP opposite a lesion site, but does not carry out subsequent polymerization (44–48). During bypass by *S. cerevisiae* Rev1, the lesion on the template strand is flipped into an extra-helical position and stabilised inside a hydrophobic pocket of Rev1, where it remains during incorporation of the incoming cytosine (49). The R324 side-chain of Rev1 displaces the DNA lesion, acting as an alternative template for Watson-Crick base pairing with the incoming cytosine (49). Following phosphodiester bond formation coupled with the hydrolysis of pyrophosphate, hydrogen bonding between the cytosine and R324 is broken (49). Rev1 then dissociates from the DNA and the lesion is reincorporated into the double helix (49).

Y-family DNA polymerases are characterised by a more open active site that can accommodate altered bases, and by the absence of 3’->5’ proofreading exonuclease activity (9, 40, 50, 51). For example, the active site of human Pol η can accommodate the two covalently-linked thymine bases in a UV-induced *cis-syn* thymine-thymine CPD lesion (50, 51). A β-strand in the little finger (LF) domain of the protein provides a molecular splint that stabilises the newly-synthesized double-stranded DNA into a B-form structure, preventing CPD-induced duplex distortion and frameshift formation, which facilitates efficient and accurate Pol η-mediated bypass of thymine-thymine CPDs (40, 50, 51).

As noted above, the capacity of TLS polymerases to accommodate altered bases in the active site, and the absence of 3’ to 5’ exonuclease activity facilitate lesion bypass (**Table 1**). Bypass is often at the cost of replication fidelity (70). The accuracy of TLS polymerases is lesion-dependent, such that specific TLS polymerases are more accurate than others when encountering particular lesions (70). For example, Pol θ predominantly incorporates the correct base when replicating across a 1,N^6^-ethenodeoxyadenosine lesion in human cells (66). However, Pol θ also plays an important role in the error-prone bypass of UV-induced *cis-syn* thymine-thymine CPDs and (6–4) PP lesions (66). By preventing the collapse of arrested replication forks and thereby reducing genome instability, error-prone lesion bypass by Pol θ protects against UV-induced skin cancer in mice (66). The overall fidelity of lesion bypass during TLS results from a combination of the biochemical properties of the individual TLS polymerases, the affinity of the polymerases for the lesion, as well as the sequence context of the lesion (9, 71).

**Table 1:** Examples of lesion bypass by human TLS polymerases.

TLS polymerase	Gene Name	Lesions bypassed
Rev1	*REV1*	UV-induced lesions (52)
		8-oxoguanine (8-oxoG) (53)
		Trans-anti-benzo[a]pyrene-N 2-dG (53)
		1,N 6-ethenoadenine adducts (53)
Pol η	*POLH*	UV-induced lesions, particularly T-T CPDs (54)
		*N*-2-acetylaminofluorene (AAF)-modified guanine (54)
		Cisplatin-induced guanine-guanine intrastrand adducts (54)
		8-oxoG (55) Abasic sites (56)
Pol ι	*POLI*	N2-guanine adduct (57)
		5-hydroxycytosine (5-OHC) (58)
		5-hydroxyuracil (5-OHU) (58)
		5,6-dihydrouracil (5,6-DHU) (58)
		8-oxoG (58)
		T-T (6–4) PP (59)
Pol κ	*POLK*	Thymine glycol (60)
		Benzo[a]pyrene-guanine adducts (BP-G) (61)
		8-oxo-dG (62)
		Acetylaminofluorene-modified G (62)
		Abasic site (63)
Pol θ	*POLQ*	Abasic sites (64)
		Thymine glycols (65)
		1,N 6-ethenoadenine adducts (66)
		UV-induced lesions (66)
Pol ζ	*REV3*	T-T (6–4) PP (67, 68)
		CPD (68)
		Extender polymerase for numerous lesions
PrimPol	*PRIMPOL*	AP site (69)

UV, ultraviolet; CPD: cyclobutane pyrimidine dimers; T-T 6-4 PP, thymine-thymine 6-4 photoproducts; XP-V, xeroderma pigmentosum variant; AP site, apyrimidinic/apurinic site.

## Homology-Directed DDT

In addition to the error-prone TLS pathway, lesion bypass during S-phase can occur through the error-free homology-directed DDT pathways, FR and TS. Error-free DNA damage tolerance requires PCNA polyubiquitination, mediated by the recruitment of one of the yeast RAD5 homologues, SNF2 histone linker PHD RING helicase (SHPRH) or helicase-like transcription factor (HLTF), to the RAD6/RAD18 complex (2). In the FR pathway, remodeling of the stalled replication fork into the characteristic ‘chicken-foot’ structure (**Figure 1**) is initiated by the recruitment of the helicase protein SMARCAL1, which binds directly to ssDNA and removes bound RPA (5, 72–74). Following the removal of RPA, translocase zinc finger RANBP2-type containing 3 (ZRANB3) then promotes further fork reversal (5, 75–77). Binding of the Fanconi anemia complementation group M (FANCM) helicase to the protein-DNA complex leads to the formation of a four-way junction (78, 79). The reversed fork is stabilized by BRCA1, BRCA2 and RAD51, which bind to the exposed ends of the nascent leading and lagging strands and prevent MRE-11-mediated exonucleolytic degradation (5, 80–83). Following successful lesion bypass, regression of the reversed fork from a four-way junction into the original three-way junction (**Figure 1**) is catalysed by RecQ-like helicase (RECQ1), Werner syndrome RECQ-like helicase (WRN) and DNA replication helicase/nuclease 2 (DNA2) (5, 84, 85).

In the TS pathway (**Figure 1**), following polyubiquitination of PCNA, the 9-1-1 complex binds to the 5’ end of the gap on the nascent DNA strand and recruits exonuclease 1 (EXO1) (18, 86). A RAD51-ssDNA presynaptic filament, stabilized by RAD55/RAD57, then forms on the ssDNA region of the template strand (87–89). ATP-dependent DNA helicase SRS2 (SRS2) disrupts the nucleofilament and opposes the action of RAD55/RAD57; the balance between these processes determines the overall stability of the RAD51-ssDNA presynaptic filament (18, 90, 91). The nucleofilament, with RAD52 and RAD54, carries out both the homology search and strand invasion of the sister chromatid (18, 89, 92). After complementary base-pairing between the invading strand and the homologous template, DNA pol δ is recruited and continues DNA replication, generating a D-loop and subsequently a sister-chromatid junction (SCJ) (18, 89, 93–95). D-loop formation is negatively regulated by SRS2 (18, 96). Finally, the slow growth suppressor 1 (SGS1)/DNA topoisomerase 3 (TOP3)/RECQ-mediated genome instability protein 1 (RMI1) complex pries the SCJ apart, regenerating the normal double-helical DNA structure (16, 18, 97, 98) (**Figure 1**).

## Regulation of DDT Pathway Choice

The type of PCNA ubiquitination plays a key role in the choice of DDT pathway between either TLS or homology-directed DDT (5, 9, 99). PCNA monoubiquitination leads to TLS, while polyubiquitination results in the initiation of homology-directed DDT. The overall level of ubiquitinated PCNA is also regulated by ubiquitin-specific processing protease 7 (USP7), by the USP1/upstream activation factor (UAF1) complex, and by enhanced level of genomic instability 1 (ELG1) (99–103). Following UV irradiation, USP1 undergoes auto-cleavage and degradation, increasing the level of modified PCNA (104). It has been proposed that the extent of replication arrest is a factor in determining the type of PCNA ubiquitination, such that prolonged replication arrest leads to polyubiquitination of PCNA molecules that remain bound at the arrest site, promoting a switch to homology-directed DDT (99). Alternatively, homology-directed DDT could be activated first where HLTF is recruited together with the RAD6/RAD18 complex, resulting in the immediate polyubiquitination of PCNA (99). In addition to ubiquitination, PCNA undergoes other related modifications. Protein inhibitor of STAT (PIAS1 and PIAS4)-mediated SUMOylation of PCNA on K164 promotes template switching rather than TLS (105). After TLS is completed, monoubiquitinated PCNA is modified by the addition of interferon-stimulated gene 15 (ISG15) molecules, leading to recruitment of USP10 and PCNA deubiquitination (99, 106, 107). Understanding the interplay between PCNA modification and the choice of DNA damage tolerance pathway is an important area for further study.

## Regulation of TLS

Since TLS polymerases are generally error-prone it is critical that TLS activity is tightly regulated. The main points of regulation of TLS involve the interactions between TLS polymerases, accessory proteins, RAD18 and PCNA (108). CHK1 and CLASPIN are essential for binding of RAD18 to chromatin (18, 109). SIVA1, TIMELESS and HLTF play important roles in PCNA monoubiquitination (18, 109–111), while protein with SprT-like domain at the N-terminus (SPARTAN) is crucial both for binding of RAD18 to chromatin and for monoubiquitination of PCNA (112, 113).

Regulation of the TLS pathway also occurs at the level of the individual polymerases, where TLS polymerases undergo post-translational modification including ubiquitination, SUMOylation and phosphorylation (as shown in **Table 2** for human Pol η). In the absence of DNA damage in the template, or when DNA lesions have been bypassed, TLS polymerases are monoubiquitinated, switching the protein from an open conformation, where the C-terminal ubiquitin-binding domain is available to interact with monoubiquitinated PCNA, to a closed conformation, where this domain is bound in *cis* to ubiquitin and is unable to interact with PCNA (128). For human Pol η, in the closed conformation ubiquitination of one of the four lysines K682, K686, K694 and K709 results in interaction between the ubiquitin moiety and the UBZ domain of the polymerase which competes with ubiquitinated PCNA for UBZ binding, thereby abrogating the PCNA interaction (118, 128). Following UV-induced DNA damage in non-small cell lung and colon carcinoma cell lines, ubiquitinated Pol η is polyubiquitinated by mouse double minute 2 homologue (MDM2) resulting in degradation by the proteasome by 24 hours post-irradiation (121, 128). The other Y family TLS polymerases Rev 1, Pol ι and Pol κ are also ubiquitinated (51, 128). In addition to ubiquitination, Pol η undergoes SUMOylation (126, 127). PIAS1-dependent SUMOylation on K163 targets Pol η to difficult-to-replicate regions of the genome such as fragile sites even in the absence of exogenous DNA damage (127). Following completion of TLS, SUMOylation of Pol η on multiple lysine residues prevents ongoing interaction with ubiquitinated PCNA, leading to SUMO-targeted ubiquitin ligase (STUbL)-mediated ubiquitination of Pol η and its’ exclusion from damage sites (127).

**Table 2:** Proteins regulating Pol η function in TLS.

Regulatory protein	Function
ATR	Phosphorylates Pol η on serine 601 and releases it from PDIP38 (114, 115)
NBS1	Binds to RAD18 and facilitates recruitment of Pol η to DNA damage sites (18, 116, 117)
SIVA1	Binds to PCNA to facilitate RAD18 recruitment and Pol η focus formation (18, 110)
SPARTAN	Binds to RAD18 and prevents its removal from DNA (18, 112, 113)
HLTF	Required for recruitment of Pol η (18, 111)
PirH2	Facilitates monoubiquitination of Pol η (39, 118, 119)
USP7	Deubiquitinates Pol η and allows it to bind to PCNA to initiate TLS (39, 120)
MDM2	Polyubiquitinates Pol η and marks it for degradation (39, 121)
PAF15	Removal of ubiquitinated PAF15 allows PCNA to bind to Pol η (39, 122); terminates TLS by removing Pol η from PCNA (39)
PARP10	Facilitates monoubiquitination of PCNA (39, 123)
CHK1, CLASPIN and TIMELESS	Promote binding of RAD18 to PCNA (39, 109)
SART3	Facilitates the binding of RPA to ssDNA and the interaction between Pol η and RAD18 (39, 124)
CDK2	Phosphorylates Pol η and increases its stability (39, 125)
PIAS1	SUMOylates Pol η at K163 to promote recruitment to replication forks (126); SUMOylates Pol η at multiple sites to target it for removal from PCNA (127)
STUbL	Extracts Pol η from DNA damage sites (127)

SPARTAN, Protein with SprT-like domain at the N terminus; HLTF, helicase-like transcription factor; PirH2, p-53 induced RING-H2 protein; USP7, ubiquitin carboxyl-terminal hydroxylase 7; MDM2, mouse double minute 2 homologue; PAF15, PCNA-associated factor 15; PARP10, poly (ADP-ribose) polymerase 10; SART3, squamous cell carcinoma antigen recognized by T Cells 3; CDK, cyclin-dependent kinase; PIAS1, Protein Inhibitor of Activated STAT 1.

TLS polymerase activity is also modulated by phosphorylation. Pol η is phosphorylated at a number of sites in the C-terminus by protein kinases including ATR, CDK2 and PKC. Following DNA damage, ATR-mediated phosphorylation of Pol η on serine 601 (114) releases it from sequestration by Pol δ-interacting protein of 38 kDa (PDIP38), freeing Pol η to bind to monoubiquitinated PCNA (115). This links ATR activation by replication arrest-induced single-stranded DNA, with recruitment of TLS polymerases to the arrested fork (115). Pol η is additionally phosphorylated by PKC on S587 and T617 (129), and on serine 687 by CDK2, which increases the stability of the polymerase in late-S and G2/M (125).

In addition to post-translational modification of specific proteins, TLS is regulated at the transcriptional level. Following DNA damage, *POLH* expression is p53-dependent (130), while *POLK* expression is regulated by the aryl hydrocarbon receptor (AhR) (131, 132). A recent report shows that TLS is negatively regulated by Pumilio RNA Binding Family Member 1 (PUM1), a protein that mediates mRNA decay (133). miRNAs have also been identified which modulate expression of DNA damage tolerance proteins. Examples include MiR-145 and miR-630 which downregulate RAD18 expression, and miR-93 and miR-619 which downregulate Pol η expression (39, 134). Furthermore, alternative polyadenylation of the *POLH* mRNA transcript in lung and bladder cancer cells generates three transcripts having 3’-UTR sequences of 427, 2516 or 6245 nucleotides, respectively (135). Of note, miR-619 only targets the longer transcript, while the shortest transcript is resistant to miR-619, and is responsible for increased Pol η expression and cisplatin resistance in cancer cell lines (135).

## Regulation of Homology-Directed DDT

Interplay between fork-protective and fork-degradative factors plays a key role in modulating fork reversal (104). BRCA1, BRCA2 and RAD51 shield the nascent DNA strands at the reversed fork from degradation by the exonuclease action of MRE-11 (5, 80, 81). WRN helicase interacting protein 1 (WRNIP1) also protects reversed forks from structure-specific endonuclease subunit SLX4 (SLX4)-mediated fork cleavage and subsequent DNA2-mediated fork degradation (136–138). The interaction of polyubiquitinated PCNA with ZRANB3 slows fork progression, promoting fork reversal through the translocase activity of ZRANB3 (75, 76). Recruitment of SMARCAL1 to the stalled fork is regulated by ATR-mediated phosphorylation, thereby limiting the extent of fork reversal (136, 139). Poly(ADP-ribose) polymerase 1 (PARP1) modulates fork reversal and fork restart by inhibiting RECQ1 helicase, and prolongs FR by preventing RECQ1-mediated regression of reversed forks to three-way junctions (15, 85, 136).

Template switching is regulated at a number of points including PCNA polyubiquitination, the formation of the RAD51-ssDNA presynaptic filament and SCJ formation [reviewed in (18)]. The chromatin remodeling protein INO80, and the human Rad5 orthologues, HLTF and SHPRH are important for PCNA polyubiquitination (140). Chromatin remodeling by INO80 facilitates the addition of K63-linked polyubiquitin chains to PCNA by HLTF and SHRPH (5, 23, 141–143). The stability of the RAD51-ssDNA filament involved in homology searching is negatively regulated by SRS2 (18, 144, 145), while exonuclease 1 (EXO1), INO80 and high mobility group protein 1 (HMO1) facilitate SCJ formation (18, 23, 89, 146).

## DNA Damage Tolerance and Carcinogenesis

The role of TLS in preventing cancer is clearly demonstrated in the sun-sensitive skin cancer-prone disease xeroderma pigmentosum variant (XP-V), where the absence of Pol η as a result of inactivating mutations in *POLH* (147, 148) leads to prolonged replication arrest at the sites of UV-induced lesions in the template. In the absence of error-free bypass of UV-induced CPDs by Pol η in XP-V cells, error-prone lesion bypass is carried out by polymerases including Pol ι and Pol ζ, resulting in increased mutagenesis that contributes to skin carcinogenesis in XP-V patients (9, 149). However, error-prone TLS can also play an anti-carcinogenic role. As noted, error-prone bypass of UV-induced lesions by Pol θ protects against skin cancer in mice, by allowing ongoing DNA synthesis to proceed thereby preventing strand break formation and the resulting genomic rearrangements (66). As a source of spontaneous mutagenesis, low-fidelity TLS polymerases may play a role in driving carcinogenesis. Y-family TLS polymerases in particular have been implicated as a source of somatic mutations in tumors (150). For example, Pol η mutational signatures are found in the genome of cancer cells from patients with malignant B-cell lymphoma and chronic lymphocytic leukemia (151).

Polymorphisms in genes encoding TLS polymerases are also associated with increased cancer risk. Polymorphisms in *REV1* and *POLI*, leading to single amino acid substitutions in Rev1 and Pol ι, were associated with increased risk of squamous cell carcinoma and adenocarcinoma, respectively (152), while *POLH* polymorphisms are associated with increased risk of malignant melanoma (153). In addition to polymorphic variants, sequencing of tumor DNA has revealed somatic mutations in TLS polymerase genes in a number of tumor types (10, 154). While the functional significance of most of these mutations remains to be determined experimentally, mutations in TLS genes that affect protein function could in principle lead to genome instability and contribute to tumor development, or alter the response of tumor cells to chemotherapeutic DNA damaging agents (10).

TLS polymerases are overexpressed in a number of different cancers. It has been proposed that overexpression of TLS polymerases can facilitate error-prone replication and adaptation of the cancer cells to targeted therapy (155). For example, expression of TLS polymerases ι, κ, λ, μ and Rev1 was upregulated in colorectal cancer cells following treatment with inhibitors of B-RAF or EGFR signalling (155). However, whether increased levels of TLS polymerases directly contribute to the acquisition of adaptive mutations requires further investigation. In non-small-cell lung tumors increased expression of Pol η is associated with poorer prognosis (156, 157), while increased expression of Pol ι is associated with oesophageal squamous cell cancer and directly correlates with the degree of metastasis (158). Pol ι expression also correlates with the grade of bladder tumors (159), while high expression of Pol κ in glioblastoma tumors is associated with poor prognosis (160).

From the perspective of cancer treatment, TLS can increase the tolerance of cancer cells to DNA damage induced by chemotherapeutic anti-cancer agents, thus promoting cancer cell survival, and increasing the mutational burden as result of error-prone lesion bypass. Pol κ plays a role in the response to the alkylating agent temozolomide used in the treatment of glioblastoma. Increased expression of Pol κ enhanced the resistance of human glioblastoma cell lines to temozolomide while down-regulation sensitised the cells to the drug (135). Pol η can bypass cisplatin-induced intrastrand lesions (161–163), and also plays a role in interstrand crosslink repair (164, 165). Human cells lacking Pol η are more sensitive to platinum-based chemotherapeutic agents (162, 163, 166–168). Overexpression of Pol η and Pol ζ contributes to cisplatin resistance in ovarian cancer stem cells and human glioma cells (169, 170). It was recently shown that PrimPol enhances survival of cisplatin-treated BRCA-deficient human ovarian cancer and osteosarcoma cells (171). PrimPol promotes repriming by reinitiating DNA synthesis downstream of blocking lesions in the template, thereby preventing fork reversal and degradation (171). In addition to promoting resistance to direct DNA-damaging agents, TLS polymerase levels also affect the response to signalling pathway inhibitors. Pol κ increased the resistance of melanoma cells to the B-RAF inhibitor vemurafenib (70). Although the mechanisms of TLS polymerase overexpression in cancer cells remain to be elucidated, overexpression of Pol κ is regulated through activation of the aryl hydrocarbon receptor (AhR) by the endogenous tryptophan-derived ligand kyneurin, as well as by DNA damaging agents such as benzo[a]pyrene (B[a]P) (131, 132, 172, 173). In the case of Pol η, expression is regulated in a p53-dependent manner after exposure of cells to DNA damage (130).

Other than DNA polymerases, alterations to regulatory proteins that play a role in TLS may also contribute to cancer development. For example, *RAD18* deletions were identified in 5% of pancreatic tumors and 11% of renal cell carcinoma tumors examined (174). Increased expression of RAD18 in a variety of human cancer cell lines (including H1299 non-small cell lung carcinoma cells, H157 and H650 adenocarcinoma cells and U2OS osteosarcoma cells) leads to excessive activation of the TLS pathway, contributing significantly to hypermutability (150). RAD18 protein levels can be increased by upregulation of melanoma antigen-A4 (MAGE-A4), which binds to and stabilises RAD18, activating the TLS pathway (175). The RAD5 ortholog HLTF, important in PCNA polyubiquitination, is downregulated through promoter methylation in colon cancer cell lines and in primary tumors (176).

## DDT Pathways as Therapeutic Targets

Given the role of DNA damage tolerance pathways in driving chemoresistance, there is potential to sensitize cancer cells to chemotherapy by inhibiting these pathways (177). To date, the major focus has been on identification of TLS inhibitors. A number of inhibitors of TLS-mediated lesion bypass have been reported (**Table 3**) and are discussed below. The inhibitors fall broadly into two categories: (i) inhibitors that directly interfere with TLS polymerase catalytic function and (ii) inhibitors that interfere with protein-protein interactions (PPIs) to inhibit TLS indirectly.

**Table 3:** Inhibitors of TLS polymerases.

Inhibitor	TLS polymerase(s)	Effect on cancer cells
Candesartan cilexetil	Pol η, Pol ι, Pol κ	Sensitises XP-V cells to UV radiation (178)
Manoalide; MK-886	Pol κ	Inhibit Pol κ *in vitro* but do not sensitise XP-V cells to UV radiation (178)
Cholesterol hemisuccinate	Pol η, Pol ι, Pol κ	Not reported (179)
*Penta*-1,2,3,4,6-O-galloyl-*beta*-D-glucose	Pol η, Pol ι, Pol κ	Not reported (180)
Pinophilins A and B	Pol η, Pol ι, Pol κ	Inhibit proliferation of cancer cell lines (181)
β-Sitosteryl (6’-O-linoleoyl)-glucoside	Pol η, Pol ι, Pol κ	Not reported (182)
3-O-methylfunicone	Pol ι, Pol κ	Inhibits cervical and colon carcinoma cell growth; sensitises cervical carcinoma cells to UV radiation (183)
Penicilliols A and B	Pol η, Pol ι, Pol κ	Not reported (184)
PNR-7-02	Rev 1, Pol η	Sensitises chronic myeloid leukaemia and ovarian cancer cell lines to cisplatin (185)
IAG-10	Pol κ	Sensitises glioblastoma cell lines to temozolomide (186)
JH-RE-06	Rev1	Sensitises melanoma cells to cisplatin; reduces melanoma tumor volume in mouse model (177, 187)
Novobiocin	Pol θ	Synthetic lethality with olaparib in HR-deficient ovarian cancer cells; tumor regression in mouse model (188)
ART558; ART812	Pol θ	Synthetic lethality with olaparib in HR-deficient colon cancer cells; inhibition of HR-deficient tumor xenografts in rat model (189)

## Direct Inhibitors of TLS Polymerases

In recent years, several TLS polymerase inhibitors have been reported (187). Examples include indole thiobarbituric acid (ITBA) and its’ derivatives (185, 186). ITBA binds directly to the finger and LF domains of Pol η, which may prevent the polymerase from binding to ssDNA and interfere with nucleotide incorporation (186). The ITBA derivative ITBA-12 inhibits Pol η and Pol κ activity (186), while ITBA-16 and ITBA-19, containing N-1-naphthoyl and N-2-naphthoyl Ar-substituents have increased specificity towards Pol η (186). The ITBA derivative, PNR-7-02 which binds to the little finger domain and inhibits Pol η function, acts synergistically with cisplatin to kill chronic myeloid leukaemia and ovarian cancer cell lines (185). An indole-aminoguanidine analogue, IAG-10, binds human Pol κ preventing the N-clasp domain from holding the LF domain in place, triggering a conformational change that decreases the contact between the protein and the DNA template (190). IAG-10 synergises with temozolomide to kill glioblastoma cell lines in culture (190). This supports the concept that direct inhibitors of TLS polymerases can increase the cytotoxic effects of conventional chemotherapeutic agents (190).

In addition to the identification of novel compounds that inhibit TLS polymerases, certain existing small molecules in clinical use have been reported to inhibit TLS. These include candesartan cilexetil, used clinically as an angiotensin-II receptor antagonist in the treatment of hypertension (191); manoalide, a phospholipase A2 inhibitor with both analgesic and anti-inflammatory properties (192), and MK-886, a leukotriene antagonist (193). Candesartan cilexetil, manoalide and MK-886 were shown to inhibit the *in vitro* activity of purified human Pol κ on undamaged DNA templates and to inhibit bypass of a γ-HOPdG lesion by Pol κ (178). Candesartan cilexetil, but not the other two compounds, sensitised Pol η-deficient XP-V cells to UV radiation (178). The fungal-derived molecules 3-O-methylfunicone, and Penicilliols A and B have been identified by screening for natural products that inhibit TLS polymerases (183). 3-O-methylfunicone, isolated from an Australian sea salt fungal strain, inhibits Y-polymerases κ, ι and η (183), competing directly with the DNA template-primer for interaction with the DNA binding domain of Pol κ (183). 3-O-methylfunicone decreased the growth of two cervical carcinoma and colon carcinoma cell lines, while having little effect on the growth and proliferation of normal human cells (183). Penicilliols A and B, isolated from a strain of *Penicillium daleae*, also inhibit mammalian Y-family polymerases, in particular Pol ι (184).

Recent reports (188, 189) demonstrating that novel inhibitors of human Pol θ synergise with HR-deficiency or resistance to PARP inhibition to kill cancer cells provides strong support for the strategy of targeting specialised DNA polymerases in cancer therapy. The Pol θ inhibitors include the antibiotic novobiocin (188) and the synthetic small molecule allosteric inhibitor ART558 (189). When used in conjunction with PARP inhibitors, Pol θ inhibitors induce synthetic lethality in HR-deficient cancer cells. PARP is required for repair of single strand breaks and inhibition of PARP-dependent single-strand break repair increases the level of double-strand breaks in the genome. Novobiocin synergistically increased the cytotoxic effects of the PARP inhibitors rucaparib and olaparib in BRCA1-deficient human retinal pigment epithelial cells and ovarian cancer cell lines, respectively (188). In mouse studies, novobiocin sensitized tumors arising from PARPi-resistant ovarian carcinoma cells to treatment with olaparib, resulting in tumor regression (188). The novel small molecule ART558 induced synthetic lethality in PARPi-resistant BRCA2-deficient human colon cancer cells treated with olaparib (189). ART812, a more bioavailable derivative of ART558, inhibits tumor xenograft growth in a rat model (189). Mechanistically, the cytotoxic effects of the Pol θ inhibitors are synergistic with HR-deficiency and PARP inhibitor resistance due to the effect of the molecules on Pol θ activity in the Theta-mediated end-joining pathway of DNA double-strand break repair (188, 189).

## Inhibitors of TLS Polymerase PPIs

Protein-protein interactions (PPIs) play a critical role in lesion bypass. PPIs include the key interactions between Ub-PCNA and TLS polymerases, as well as interactions between inserter and extender TLS polymerases, for example between Rev1 and other TLS polymerases, and between the Rev7 and Rev3 subunits of the Pol ζ complex. A number of PPI inhibitors have been developed based on detailed structural information on the interaction domains of the target proteins (194).

## Inhibitors of Interactions Between PCNA and TLS Polymerases

Indirect inhibitors of TLS can inhibit the recruitment of TLS polymerases to PCNA, thereby preventing lesion bypass. Small molecule inhibitors of the PCNA/PIP-box interaction compete with the PIP-box sequence of TLS polymerases for binding to PCNA during the initiation phase of TLS. The compound 3,3’,5-triiodothyronine (T3) and its synthetic derivative T2 amino alcohol (T2AA) were reported to inhibit the PCNA/PIP-box PPI (195). T2AA and its analogues suppressed TLS in NER-deficient human cells, decreased cell division in osteosarcoma cells treated with cisplatin (196), and inhibited interstrand DNA cross-link (ICL) repair, slowing proliferation of cervical cancer cells (196). Consistent with the importance of the PCNA-PIP box interaction, a novel compound which specifically targets the L126 to Y133 region of the PIP-interaction loop of PCNA sensitises cancer cells to cisplatin (197). Other inhibitors of the PCNA/TLS polymerase interaction specifically prevent the recruitment of Rev1 to PCNA. One small molecule inhibitor, compound 1, binds Rev1 directly *via* the UBM2 motif and prevents interaction with K164-ubiquitinated PCNA (198). Compound 1 increased the cytotoxicity of both 4-hydroxycyclophosphamide and cisplatin by up to 10-fold in cultured cells (198).

## Inhibitors of PPIs Between Inserter and Extender Polymerases

Inhibitors of essential PPIs between inserter and extender TLS polymerases have also been identified that suppress TLS and enhance the cytotoxicity of chemotherapeutic agents. Among these are small molecule inhibitors of the interaction between the C-terminal domain of Rev1 (Rev1-CT) and the Rev1-interacting region (RIR) of other Y-family TLS polymerases. Two such compounds, 4 and 5, have been reported (199) that bind to the Rev1-CT, preventing the recruitment of Pol ζ to Rev1, and sensitising fibrosarcoma cell lines to cisplatin and to UV radiation. Both compounds decreased the level of cisplatin-induced *HPRT* gene mutations, indicating the molecules can attenuate the mutagenicity associated with error-prone replication of platinum-induced DNA lesions (199). Additional derivatives have been developed that also compete with TLS polymerases for binding to the Rev1-CT domain, and increase the cytotoxicity of cisplatin (200, 201). Of note, inhibition of the Rev1-CT/RIR interaction was synergistic with the ATM and ATR inhibitor VE-821 and the Wee1 inhibitor MK-1775, leading to the formation of daughter strand gaps (DSGs) in replicating DNA, and sensitising bone osteosarcoma and colon cancer cells to these agents (201). Consistent with this, Pol η-deficient cells are significantly more sensitive to ATR inhibitors than normal cells (129, 202, 203). Since daughter strand gaps can also result from replication stress in oncogene-mutated cancer cells, it is proposed that in addition to direct lesion bypass, TLS polymerases contribute to cancer cell survival by carrying out DNA synthesis at DSGs. This limits the accumulation of single-stranded DNA in the genome (201), a process termed gap suppression (201). TLS inhibitors could therefore be used to achieve synthetic lethality in combination with cell cycle checkpoint inhibitors that induce DSGs, such as inhibitors of ATR and Wee1 (201).

Aside from inhibitors that interrupt Rev1-CT-RIR interactions, a small molecule inhibitor that disrupts the interaction between Rev1 and the Rev7 subunit of Pol ζ has also been identified. In both human and mouse cell lines, JH-RE-06, a small molecule that binds to the C-terminal domain of Rev1 and blocks interaction with the Rev7 subunit of Pol ζ, sensitised melanoma cells to cisplatin, and reduced drug-induced mutagenesis (204). Combination treatment with JH-RE-06 and cisplatin reduced tumor volume and improved survival in a mouse xenograft model of A375 melanoma cells, demonstrating the potential of targeting key PPIs as a therapeutic strategy (204, 205). Treatment of fibrosarcoma and melanoma cells with JH-RE-06 leads to senescence following cisplatin-induced DNA damage (204, 205). The impact of chemical inhibition of Pol ζ activity on overall genome stability should also be considered, since genetic ablation of *REV3L* encoding the catalytic subunit of Pol ζ increased genome instability in *REV3L*-null mouse embryo fibroblasts (206), and contributed to development of lymphomas and mammary tumors in mice where *REV3L* was conditionally deleted (207).

Recent evidence indicates that the interaction between Rev7 and Rev3 proteins required to form active Pol ζ is actively regulated in cells (208). The ATPase thyroid receptor-interacting protein 13, (TRIP13), modulates the conformation of Rev7, preventing both its’ interaction with Rev3 to form of active Pol ζ which is required for TLS, and interaction with the Shieldin complex which activates NHEJ (208). TRIP13 therefore mediates pathway choice, promoting error-free HDR over mutagenic TLS or NHEJ (208). TRIP13 overexpression correlates with BRCA1-deficiency in breast cancer cells and contributes to chemoresistance towards PARP inhibitors (208). The interaction of Rev7 with the Shieldin complex and with Rev3 is also inhibited by expression of the p31^comet^ HORMA-like protein (209, 210), identifying Rev7 as a key modulator of pathway choice after DNA damage. Overall, inhibition of key interactions between TLS polymerases and partner proteins represents a promising approach to sensitising cancer cells to chemotherapy.

## Inhibitors of TLS Regulators

While targeting the catalytic activity and protein-protein interactions of TLS polymerases has been shown to be effective in overcoming chemoresistance in cancer cells, there may also be potential in targeting upstream regulators of TLS to inhibit the action of multiple TLS polymerases for therapeutic effect. For example, targeting the TLS pathway regulator RAD6 and PCNA monoubiquitination could be a more potent method to inhibit TLS than targeting individual TLS polymerases. However, the effects of inhibiting RAD6 on cytotoxicity and genome stability after DNA damage should be directly compared with the effects of targeting individual TLS polymerases using specific inhibitors. Inhibition of RAD6 by a small-molecule inhibitor, SMI#9, attenuated cisplatin resistance in triple-negative breast cancer cells, and enhanced the cytotoxicity of oxaliplatin towards the oxaliplatin-resistant colorectal carcinoma cell line HCT116-OxR (211, 212). Co-administration of SMI#9 with cisplatin decreased the growth of tumors arising from triple-negative breast cancer cells and lymph node metastasis (211). Molecules that modulate the extent of PCNA monoubiquitination and therefore the recruitment of TLS polymerases to DNA damage sites have also been described. C11 and G8, two inhibitors of the protein kinase AKT, inhibit damage-induced PCNA monoubiquitination and show synthetic lethality with UV-irradiation in *BRCA1*-deficient triple-negative breast cancer and colon cancer cell lines (213). The specific targets of AKT that modulate PCNA monoubiquitination are of interest (213).

Additional potential targets for inhibition of TLS include the USP1/UAF1 deubiquitinase complex. Two small molecule USP1/UAF1 inhibitors, pimozide and GW7647, enhanced the cytotoxicity of cisplatin and decreased cell division in non-small cell lung cancer cell lines (214). A third USP1/UAF1 inhibitor, ML323, was found to increase the cytotoxic effect of cisplatin on osteosarcoma and non-small cell lung cancer cells (215). As well as affecting TLS, USP1/UAF1 inhibitors increase cisplatin sensitivity by disrupting deubiquitination of the FANCD2/FANCI complex, preventing repair of drug-induced interstrand adducts by the FA crosslink repair pathway (215–217). The level of the chaperone protein Hsp90 is also important in the stability of TLS polymerases, including Rev1 (218) and Pol η (219). Tanespimycin (17-AAG), which promotes proteasomal degradation of Hsp90, decreased the amount of Rev1 in human prostate and bone osteosarcoma cancer cells (218), and downregulated the recruitment of Rev 1 to sites of UV-induced DNA damage in the nucleus (218). The proteasome inhibitors, lactacystin and MG-132, prevented the reduction in Rev1 levels induced by 17-AAG (218), indicating that Hsp90 normally protects Rev1 from proteasomal degradation. Following UV-induced DNA damage, Pol η undergoes direct PIAS1-mediated poly-SUMOylation upon recruitment to PCNA. The protein is then modified by SUMO-targeted ubiquitin ligases (STUbLs), which is crucial for clearance of the polymerase from damage sites following lesion bypass. Targeting the human STUbLs RNF4 and RNF11 could potentially enhance the turnover rate of mutagenic TLS polymerases and decrease lesion bypass in cancer cells (127). Given the extensive ubiquitination of proteins involved in DNA damage tolerance, the response to general inhibitors of the proteasome is complex. Of interest, it has been reported that the proteasome inhibitors MG-132, lactacystin, and MG-262 inhibited TLS in human cancer cell lines, but not in normal cells (220), indicating that targeting the degradation of specific proteins could represent another approach to modulation of the DNA damage tolerance pathway in cancer cells.

## Other Approaches to TLS Inhibition

Alternative approaches of TLS inhibition include the use of novel non-natural nucleotides to inhibit bypass synthesis, and down-regulation of DDT protein expression using miRNAs. Two synthetic nucleotide analogues, 5-nitro-indolyl-2′deoxyriboside triphosphate (5-NITP) and 5-phenyl-indolyl-2′deoxyriboside triphosphate (5-PhITP), are preferentially incorporated opposite abasic sites during DNA replication, while resisting both excision by proofreading exonuclease activity and subsequent elongation, preventing further DNA replication past the damage sites (221). TLS polymerases, specifically Pol η and Pol ι, preferentially inserted 5-NITP over dATP opposite temozolomide-induced abasic sites, preventing lesion bypass at the damage sites (222). The synthetic nucleoside potentiated the cytotoxic effects of temozolomide in glioblastoma cancer cell lines, and led to tumor regression in mouse models of tumor growth (222).

Identification of miRNAs that regulate expression of key genes represents another potential mechanism of DDT inhibition. For example, miR-96 regulates expression of *RAD51* and *REV1* (223). Knockdown of *REV1* expression using miR-96 contributed to cisplatin sensitization in bone osteosarcoma cells with intact HR repair, as well as in *BRCA1*-deficient breast cancer and *BRCA2*-deficient ovarian cancer cell lines with compromised HR pathways (223). Direct inhibition of *RAD51* and *REV1* expression by overexpression of miR-96 ultimately slowed growth of tumors from triple-negative breast cancer cells in mice (223).

## Future Perspectives

There is increasing evidence that inhibition of DNA damage tolerance pathways can sensitize cancer cells to conventional chemotherapeutic agents. Ongoing research into the molecular basis of DNA damage tolerance will provide opportunities for further advances. The factors that influence pathway choice in specific circumstances require further investigation. For the TLS pathway, understanding the contribution of individual DNA polymerases to lesion bypass in cancer and normal cells will be important, and will further inform the design of specific inhibitors that target polymerases or accessory proteins. In addition to polymerases, other DDT proteins could also represent therapeutic targets, using agents that directly block protein function, or interfere with specific PPIs that are essential for DNA damage tolerance. The major focus to date has been on inhibition of TLS, but given the recent advances in elucidating the genetics and biochemistry of fork reversal and template switching, there is potential to identify new inhibitors targeting these pathways. The risk of directing replication intermediates into other more error-prone pathways, increasing genetic instability and further contributing to the development of resistant cancer cells, also has to be considered in this context. Further challenges include the development of specific inhibitors, proving that cellular phenotypes are due to inhibition of the proposed target, and demonstrating clinical utility for small molecule inhibitors. Ongoing research will advance the potential to target DNA tolerance pathways as a therapeutic approach for cancer.

## Conclusion

DDT pathways are critical to allow cells to tolerate DNA lesions and facilitate the completion of DNA replication. However, imbalances in these pathways in cancer cells can lead to significant mutagenesis, contributing to chemoresistance and increased cancer cell survival. Considering the evidence that inhibiting DDT pathways can sensitize cancer cells to chemotherapy, more research into novel therapeutics in this area could eventually lead to the development of a new class of cancer therapeutic agents that enhance the response to treatment with conventional chemotherapy.

## Author Contributions

AL wrote the initial draft of the review and MC revised and edited the review. Both authors approved the final version.

## Funding

Open access publication fees were supported by the Discipline of Biochemistry, The National University of Ireland Galway.

## Conflict of Interest

The authors declare that the research was conducted in the absence of any commercial or financial relationships that could be construed as a potential conflict of interest.

## Publisher’s Note

All claims expressed in this article are solely those of the authors and do not necessarily represent those of their affiliated organizations, or those of the publisher, the editors and the reviewers. Any product that may be evaluated in this article, or claim that may be made by its manufacturer, is not guaranteed or endorsed by the publisher.

## Abbreviations

ATR, ataxia telangiectasia and RAD3-related protein; ATRIP, ATR-interacting protein; DDT, DNA damage tolerance; FA, Fanconi anemia; FR, fork reversal; HTLF, helicase-like transcription factor; PARP, Poly(ADP-ribose) polymerase; PCNA, proliferating cell nuclear antigen; PIP, PCNA-interacting peptide; PPI, protein-protein interactions; Rev1-CT, Rev1 C-terminal; RIR, Rev1-interacting region; RPA, Replication protein A; SHPRH, SNF2 histone linker PHD RING helicase; SMARCAL1, SWI/SNF-related matrix associated actin-dependent regulator of chromatin subfamily A-like protein 1; SPARTAN, SprT-like domain at the N-terminus; ssDNA, single-stranded DNA; TLS, translesion synthesis; TLSi, translesion synthesis inhibitor; TS, template switching; UBM, ubiquitin-binding motif; UBZ, ubiquitin-binding zinc finger; USP1, ubiquitin carboxyl-terminal hydroxylase 1; USP7, ubiquitin carboxyl-terminal hydroxylase 7; XP-V, xeroderma pigmentosum variant; ZRANB3, Translocase zinc finger RANBP2 type –containing 3.
